# Differentially Expressed Genes in the Cuticle and Hemolymph of the Silkworm, *Bombyx mori*, Injected with the Fungus *Beauveria bassiana*

**DOI:** 10.1673/031.013.13801

**Published:** 2013-11-30

**Authors:** Cheng-Xiang Hou, Guang-Xing Qin, Ting Liu, Xing-Lin Mei, Bing Li, Zhong-Yuan Shen, Xi-Jie Guo

**Affiliations:** 1 Sericultural Research Institute, Jiangsu University of Science and Technology, Zhenjiang 212003, Jiangsu, China; 2 Key Laboratory of Silkworm and Mulberry Genetic Improvement, Ministry of Agriculture of China, Sericultural Research Institute, Chinese Academy of Agricultural Sciences, Zhenjiang 212018, Jiangsu, China

## Abstract

The most important pathogenic fungus of the silkworm, *Bombyx mori* L. (Lepidoptera: Bombycidae), is *Beauveria bassiana* (Balsamo-Crivelli ) Vuillemin (Hypocreales: Clavicipitaceae), which causes significant damage to sericulture production. Therefore, diagnosing fungal disease and developing new control measures are crucial to silk production. To better understand the responsive and interactive mechanisms between the host silkworm and this fungus, variations in silkworm gene expression were investigated using the suppression subtractive hybridization method following the injection of *B*. *bassiana* conidia. Two cDNA libraries were constructed, and 140 cDNA clones were isolated. Of the 50 differentially expressed genes identified, 45 (112 clones) were identified in the forward library, and 5 (28 clones) were identified in the reverse library. Expression profiling of six of these genes by quantitative polymerase chain reaction (qPCR) verified that they were induced by the fungal challenge. The present study provides insight into the interaction between lepidopteran insects and pathogenic fungi.

## Introduction

*Beauveria bassiana* Balsamo-Crivelli Vuillemin (Hypocreales: Clavicipitaceae) is an entomopathogenic fungus that grows naturally in soil and has been widely used in the biological control of insects in forests. However, its mechanism of action remains elusive despite chemical and synthetic methods of analysis (Grogan and Holland 2000; Hao et al. 2001). In the natural environment, *B. bassiana* usually infects insects and causes white muscardine disease through cuticle penetration. The fungus is then fought by the innate immune responses of the insects, including cellular and humoral mechanisms (Lavine and Strand 2002; Hou et al. 2011). Entomopathogenic fungi replicate in the insect hemolymph. However, the insect genes that respond to fungal infection and replication are still not well known.

The silkworm, *Bombyx mori* L. (Lepidoptera: Bombycidae), is a typical lepidopteran insect and is economically important for silk production in many developing countries. It has also contributed enormously to the study of insect genetics and immunology (Goldsmith et al. 2005; Li et al. 2005; Liu et al. 2009).

*B. bassiana* is one of the major fungal pathogens of the silkworm and can cause enormous damage to the sericulture industry. The molecular mechanism by which it infects silkworms is still poorly understood. Therefore, elucidating the mechanism of antifungal immunity of the silkworm is important to improve its antifungal ability. The identification and study of differentially expressed insect genes after immune challenge will lead to a better understanding of the breadth and regulation of insect immune responses. Suppression subtractive hybridization (Diatchenko et al. 1996) has been used to identify immune-inducible genes in the mosquito (Oduol et al. 2000), tsetse fly (Hao et al. 2001), and *Manduca sexta* (Zhu et al. 2003). In the present study, suppression subtractive hybridization was used to identify the differentially expressed genes between *Beauveria*injected and water-injected silkworm larvae, and these data were verified by real-time quantitative polymerase chain reaction (qPCR).

## Materials and Methods

### Silkworm strain

The silkworm strain Dazao, provided by the Sericultural Research Institute of the Chinese Academy of Agricultural Sciences, was used in the study. The larvae were reared on fresh mulberry leaves at 25° C. Third-day fifth instar larvae were used in the experiments.

### Treatment with *B. bassiana* conidia

*B. bassiana* conidia were diluted to a concentration of 108 spores/mL with sterile distilled water. Each larva was injected with 1 µL of the conidia solution. The larvae of the control group were injected with the same amount of sterile distilled water. The larvae were reared under high temperature and high humidity conditions (28° C and 95% RH) for 8 hours to promote conidia germination and then returned to normal conditions (25 °C and 80% RH).

### Cuticle and hemolymph collection

After injection with the conidia and sterile distilled water, the cuticles and hemolymph of the larvae were collected at 3, 6, 9, 12, and 24 hours after injection. The hemolymph was directly mixed with pre-joined Trizol reagent (Life Technologies, www.lifetechnologies.com) in an Eppendorf tube. The cuticles were quickly washed in a diethylpyrocarbonate-treated phosphate buffered saline solution (137 mM NaCl, 2.68 mM KCl, 8.1 mM Na2HPO4, and 1.47 mM KH_2PO4_ at pH 7.4) and immediately frozen in liquid nitrogen.

### Isolation of total RNA and polyA+ RNA

The total RNA from the cuticles and hemolymph of the *B. bassiana*-injected and water-treated larvae was extracted using TRIzol reagent and subjected to DNase I (Takara Bio, www.takara-bio.com) treatment according to the manufacturer' s instructions. The total RNA concentration was determined using a Biophotometer (Eppendorf, www.eppendorf.com) by measuring the absorbance values at 260 nm and 280 nm (A260:A280). Equal amounts of total RNA from the samples of the five time points were pooled. The Oligotex mRNA Mid Kit (Qiagen, www.qiagen.com) was used to purify polyA+ RNA from the RNA pool, and 2 µg of poly(A)+ RNA was used as the starting material for reverse transcription to construct the subtracted cDNA libraries.

### Construction of cDNA libraries by suppression subtractive hybridization

Suppression subtractive hybridization was performed using a Clontech PCR-select cDNA subtraction kit (Clontech Laboratories, www.clontech.com). Reciprocal forward and reverse subtractions were performed according to the manufacturer' s instructions. The RNA extracted from the cuticles and hemolymph at all the time points was equally mixed. The forward library was constructed using the cDNA of the *B. bassiana*-injected cuticles and hemolymph as the tester and the cDNA of the control as the driver. The reverse library was constructed using the cDNA of the control as the tester and the cDNA of the *B. bassiana*-injected cuticles and hemolymph as the driver.

The subtracted cDNA libraries were generated by inserting the differentially expressed cDNA fragments into the pGEM-T Easy vector (Promega, www.promega.com) and transforming these vectors into JM109 competent cells. Aliquots (100 µL) of the transformation mixture were then spread onto Luria-Bertani agar plates containing 100 mg/mL ampicillin, 80 mg/ml X-gal, and 50 mM isopropyl 1-thio-β-D-galactopyranoside and incubated at 37° C overnight. Some subtractive clones were sequenced, and the nucleotide and amino acid sequence homologies were determined by searching the NCBI/GenBank database using the BLASTX algorithm.

### Confirmation of differentially expressed genes by real-time qPCR

The total RNA from the cuticles and hemolymph of *B. bassiana*-infected and control larvae was extracted using Trizol reagent (Life Technologies). The RNA was treated with DNase I following the manufacturer' s instructions. The concentration of DNase I-treated RNA was adjusted with H2O to 1 µg/µL, and 1 µg of the DNase I-treated RNA was reverse transcribed in a 20 µL reaction using a PrimeScript RT reagent kit (TaKaRa). Real-time qPCR was performed using 2 µL of the diluted firststrand cDNA (1/100) in each 20 µL reaction mixture. The reaction was performed with specific primers for amplifying the following genes: chemosensory protein 11, muscle LIM protein isoform 1, transferrin, arylphorin, sexspecific storage-protein SP1 precursor, lysozyme, moricin, and the low molecular lipoprotein 30K pBmHPC-6 (Lp-c6) (Table 1). The relative expression levels of each gene at different time points were normalized using the Ct values obtained for *B. mori* glyceraldehyde-3-phosphate dehydrogenase (*Bm*GAPDH), an endogenous control gene, amplification in the same plate. In each assay, the expression level is shown relative to the lowest expression level, which was arbitrarily set to one. All samples were tested in triplicate. The mean values and the standard deviations were used for the analysis of the relative transcript levels for each time point using the relative quantitative method (ΔΔCt).

Real-time qPCR was performed using 2 µL of diluted first strand cDNA (1/100) in each 20 µL reaction volume using SYBR Premix Ex Taq (TaKaRa) according to the manufacturer' s instructions. Specific primers for the genes chemosensory protein 11, muscle LIM protein isoform 1, transferrin, arylphorin, sexspecific storage-protein SP1 precursor, low molecular lipoprotein 30K pBmHPC-6 (Lpc6), lysozyme, moricin, and *Bm*GAPDH are listed in Table 1. The final concentration of each primer was 100 nM. The reactions were run in triplicate on an Opticon system (Bio- Rad, http://www.bio-rad.com) using the following thermal cycling parameters: 95° C for 5 sec and 40 cycles at 60° C for 10 sec and 72° C for 10 sec. The melting curves were constructed after amplification. The data were analyzed and normalized relative to the *Bm*GAPDH transcription levels using Opticon monitor analysis software (BioRad). The ΔΔCt method was used to evaluate the relative expression differences.

## Results

### EST sequencing and identification

The cDNA libraries, both forward and reverse, were constructed from the silkworm larvae injected with *B. bassiana* conidia using suppression subtractive hybridization. The forward library was constructed with cDNA from the *B. bassiana*-injected cuticles and hemolymph as the tester and cDNA of the control as the driver, whereas the reverse library used cDNA of the control as the tester and cDNA of the *B. bassiana*-injected cuticles and hemolymph as the driver. A total of 140 cDNA clones were isolated, 112 from the forward library and 28 from the reverse library. In total, 45 genes were identified in the forward library, and five genes were identified in the reverse library (Tables 2 and 3).

The genes present in the forward library were upregulated by *B. bassiana* infection (Table 2) and were more abundant than the genes in the reverse library (Table 3). Using blast2GO software and the annotations of the *Spodoptera frugiperda* sequences (Barat-Houari et al. 2006), the subtractive genes were classified into five groups: genes encoding proteins ubiquitously expressed by many cell types (AI-AIX), genes responsible for cell-cell communication (BI-BIII), genes encoding transcription factors and gene-regulatory proteins (C), genes encoding molecules expressed in insects (DI-DIV), and others (EI-EIII). There was only one overlapping gene between the two libraries, which encodes the *B. mori* 30 kDa lipoprotein 19G1 precursor (Tables 2 and 3).

Some upregulated genes identified in our previous study on the percutaneously infected silkworm were also induced in the present experiment, such as putative cuticle proteins, heat shock proteins, ribosomal proteins, and the antimicrobial proteins lysozyme and the cecropin B precursor. The newly identified genes in the present study included those encoding ferritin, transferrin, ATP synthase subunit, troponin, storage proteins (arylphorin, sex-specific storage protein (SP1), and sexspecific storage protein 2 (SP2)), a 30 kDa lipoprotein, and chemosensory protein 11 (CSP11). The genes that encode ferritin, trans ferrin, heat shock proteins, CSP11, and storage proteins were immune-responsive, and the genes that encode moricin and gloverin 4 were anti-microbial, whereas the genes that encode ribosomal proteins, elongation factors, and ATP synthase were involved in the pathologic processes. Furthermore, ribosomal proteins also participated in signal transduction.

### Determination of differentially expressed cDNAs by real-time qPCR

**Figure 1.**
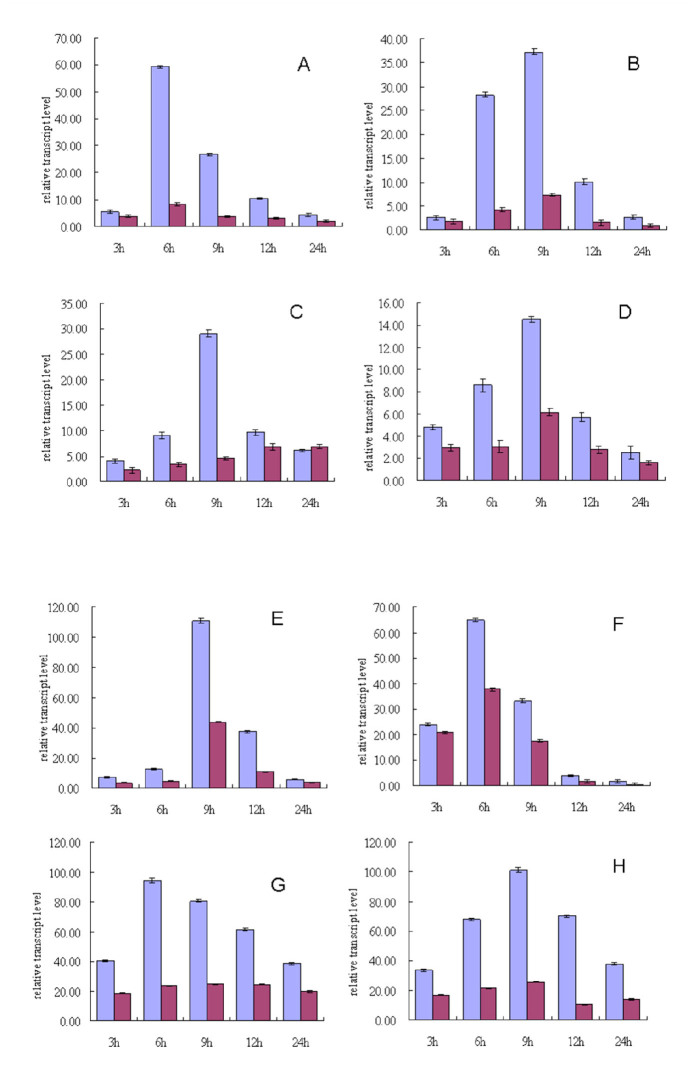
Variations in expression levels of fungus-responsive genes in the hemolymph of silkworms, *Bombyx mori*, infected with *Beauveria bassiana*. The third-day larvae of the fifth instar of Dazao strains were infected with *B. bassiana*. Total RNA was extracted from the hemolymph at the indicated time points after infection and subjected to DNase I treatment and reverse transcription. Two microliters of each 10-fold diluted first strand cDNA (20 ng) was analyzed in each real-time qPCR reaction. The reaction was performed with specific primers for amplifying each of the six genes. The relative expression level of each gene at each time point was normalized using the Ct values obtained for the *Bm*GAPDH amplifications run in the same plate. In each assay, the expression level is shown relative to the lowest expression level, which was set to one. All samples were tested in triplicate. The mean value ± SD was used for the analysis of the relative transcript levels for each time point using the △△Ct method. The *B. bassiana*injected and water-treated individuals are shown on the left (blue) and right (purple), respectively. A. Chemosensory protein 11; B. Muscle LIM protein isoform 1; C. Transferrin; D. Sex-specific storage-protein SP1 precursor; E. Arylphorin; F. Low molecular lipoprotein 30K pBmHPC-6 (Lp-c6); G. lysozyme; H. Moricin. High quality figures are available online.

The differential gene expression in the larval cuticles and hemolymph upon *B. bassiana* infection was determined via real-time qPCR (Figure 1). The present study focused on the genes that were present in the forward library, as they are likely responsive to the *B. bassiana* infection and may contribute to the resistance of the silkworm against *B. bassiana* infection and propagation. The transcript abundances of the selected eight genes expressed differently in the hemolymph between the *B. bassiana*-injected and the waterinjected larvae were compared using real-time qPCR (Figure 1).

In the hemolymph of the *B. bassiana*-injected larvae, the transcript level of the CSP11 gene was the highest at 6 hours after injection and then gradually decreased, whereas the transcription in the control larvae stayed almost constant in the same period (Figure 1A). The hemolymph transcript levels of the low molecular lipoprotein 30K pBmHPC-6 (Lp-c6) gene exhibited the same expression pattern for both *B. bassiana*-injected and water-injected larvae and reached a peak at 6 hours after injection; however, its transcript levels in the *B. bassiana*-injected larvae were much higher than those in the water-injected larvae (Figure 1F). The gene transcript levels of muscle LIM protein isoform 1, transferrin, sex-specific storage-protein SP1 precursor, and arylphorin reached their peaks at 9 hours after injection and then gradually decreased in both the *B. bassiana*-injected and the water-injected larvae. However, the transcript levels in the *B. bassiana*-injected larvae were much higher than those in the water-treated larvae, and the transcript level of the arylphorin gene was the highest among the six genes (Figure 1B–1E). In the *B. bassiana*-injected larvae, the transcript levels of lysozyme and moricin reached their peaks at 6 hours and 9 hours after injection, respectively, and then gradually decreased; however, their expression levels not significantly different from the waterinjected control. The transcription of these genes in the cuticles in the same period was also detected, but the differential expression between the *B. bassiana*-injected and waterinjected larvae was not obvious and almost negligible (data not shown).

## Discussion

The mechanisms of immune responses to bacteria in silkworms have been extensively studied. The responsive mechanisms of insects to fungal infection and some antifungal peptides have drawn the attention of researchers, and related signal transduction molecules have been identified (Belvin and Anderson 1996; Hoffmann 2003). Differential gene expression in silkworm larvae percutaneously infected with *B. bassiana* was reported in our previous study (Hou et al. 2011). To better understand the silkworm responsive mechanism to fungal infection, silkworms were infected in the present study by injecting *B. bassiana* conidia into the hemolymph of the larvae, and the genes that were differentially expressed upon infection were analyzed.

After fungal conidia enter the hemolymph of the silkworm, a series of immune changes occurs in the hemolymph (Hoffmann and Reichhart 2002; Tzou et al. 2002). The expression levels of some genes that are responsive to fungal invasion also change after fungal injection, as identified in the present study. Most of these genes are functional; their expression was upregulated, and they are likely to play important roles as the infection progresses. In the present experiment, only a few differentially expressed genes were detected in the reverse library. These results are similar to those of our previous study (Hou et al. 2011). Therefore, the subsequent analysis focused on the identification of differentially expressed genes present in the forward library because these genes are likely responsive to *B. bassiana* infection and may affect *B. bassiana* proliferation in the silkworm larvae.

The differentially expressed genes identified in the forward library were upregulated, which implies that these genes are induced by or responsive to *B. bassiana* infection. Some of these genes encode storage proteins, such as arylphorin, SP1, SP2, and some 30 kDa lipoproteins. These storage proteins are the major nutrient sources for growth and development, especially for insect metamorphosis (Willott et al. 1989; Spyliotopoulos et al. 2007). They are also constituents of the sclerotizing system of the cuticle (Dong et al. 1996). Among these storage proteins, arylphorin transcript levels were suppressed during parasitization of *Heliothis virescens* larvae by *Campoletis sonorensis* (Shelby and Webb 1997). In the fifth instar silkworm larvae, the amount of SP1 is higher females than in males ( Tojo et al. 1980; Mine et al. 1983). In the present study, the transcript levels of these storage protein genes were upregulated in both the infected and control larvae and reached their peak level at 6–9 hours after injection, although the transcript level in the infected larvae increased more sharply (Figure 1 D–F). This implies that a sharp increase in these storage proteins in the hemolymph likely contributes to the inhibition of the generation and proliferation of *B. bassiana* at the early stage of infection. The increase in the transcript level in the control larvae may contribute to sclerotization of the cuticle, which heals injuries to the exoskeleton caused by puncturing.

The muscle LIM protein and chemosensory proteins may play important roles in the early events of the recovery process of skeletal muscles to injury and in cuticle synthesis, tissue formation, and regeneration (Nomura Kitabayashi et al. 1998; Stathopoulos et al. 2002; Sabatier et al. 2003; Barash et al. 2005; Fort et al. 2007). LIM participation is required in many morphologic and histologic changes in insects during the transformation from larva to adult (Liu et al. 2007). Some chemosensory proteins might be involved in the immune response (Oduol et al. 2000; Stathopoulos et al. 2002; Sabatier et al. 2003). In the present experiments, the transcript levels of LIM and CSP11 were also upregulated by injecting either conidia or water, but the degree of upregulation was greater when condidia were injected (Figure 1A and B). This result implies that muscle LIM protein and CSP11 are not only involved in the recovery and synthesis of damaged skin but also strongly respond to *B. bassiana* invasion. However, the effect of the increase in the transcript levels on the generation of conidia and the proliferation of *B. bassiana* needs to be studied further.

Transferrin (Tf) is a multifunctional protein (Nichol et al. 2002) that possesses antimicrobial properties against bacteria and fungi (Yoshiga et al. 1997; Yun et al. 1999; Kucharski and Maleszka 2003; Thompson et al. 2003). *Bm*Tf may play an important role in pathogen clearance in insect innate immunity (Yun et al. 2009). In the present test, the expression level of the *Bm*Tf gene increased at the early stage of injection and reached its peak at 9 hours after injection (Figure 1C). Perhaps *Bm*Tf affected *B. bassiana* gene expression in the *B. mori* host cells and resulted in the inhibition of *B. bassiana* proliferation.

Compared with our previous study on cuticle infection, many storage proteins and transport proteins were present in the hemolymph when the larvae were injected with *B. bassiana*. We found that the changes in the transcription levels of genes in the cuticle were not obvious in the hemolymph by real-time qPCR. These results suggested that the fungal pathogen resistance mechanism is different between the hemolymph and the cuticle.

In conclusion, *B. bassiana* infection and the defense response in the silkworm are complex processes that involve signal recognition and transduction and the production of antifungal factors. Differentially expressed genes were identified in silkworm larvae infected with *B. bassiana*, which are probably induced by and responsive to the infection. These results may provide new insights into the complex interactions between *B. bassiana* and silkworms and may be used to gain a better understanding of the defense strategies elicited by the lepidopteran host against fungal infection. Further studies are necessary to identify the functions of these genes and their roles in the silkworm defense upon fungal infection.

**Table 1.**
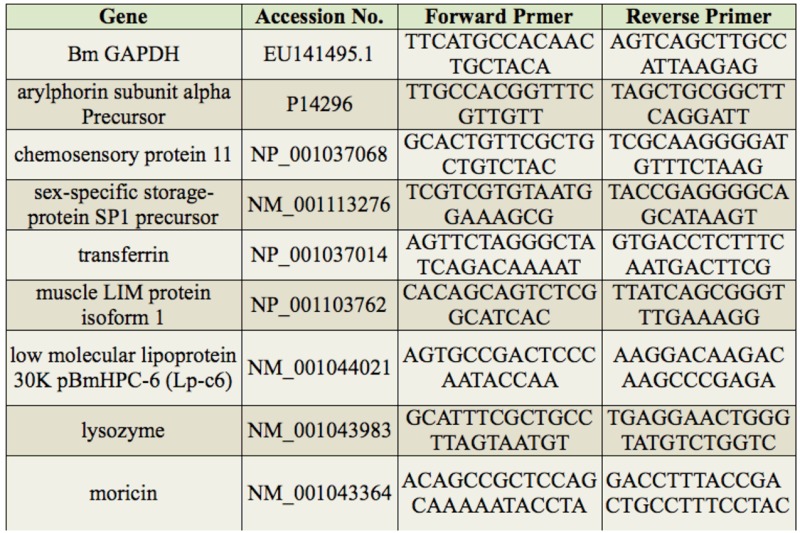
Primers used in real-time qPCR for confirmation of the differentially expressed genes.

**Table 2.**
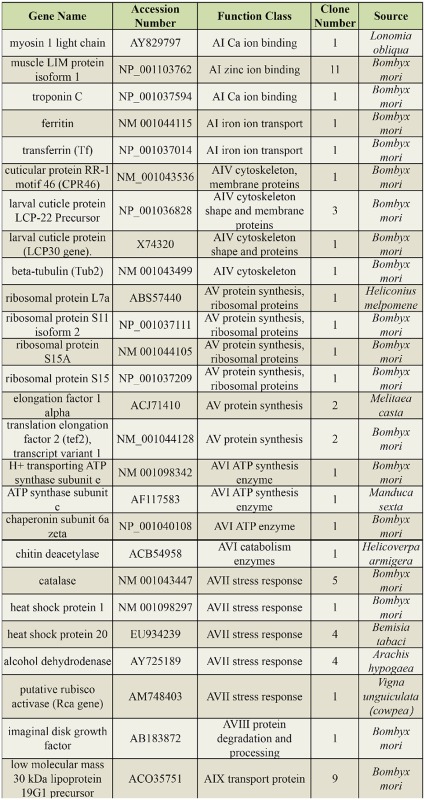
Differentially expressed genes from the forward SSH library and their major functional categories.

**Continued:**
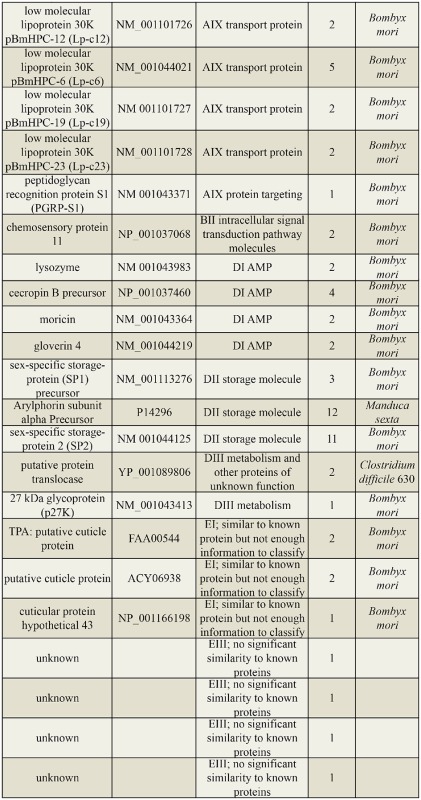

**Table 3.**
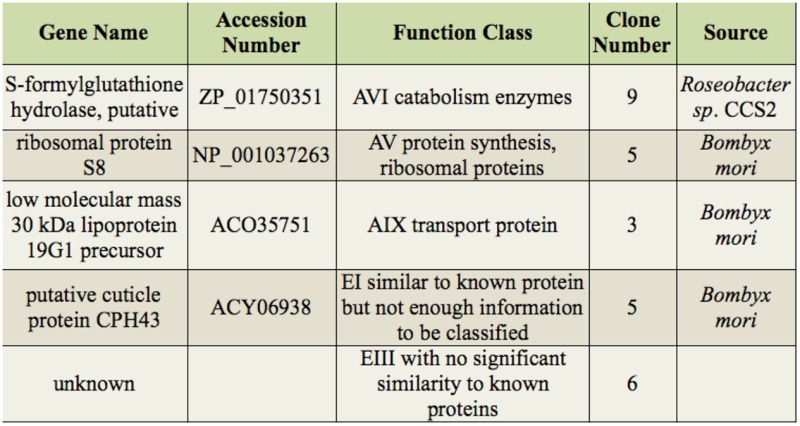
Differentially expressed genes in the reverse SSH library and their major functional categories.

## Abbreviations

*Bm*GAPDH,: Bombyx mori glyceraldehyde-3-phosphate dehydrogenase;
**qPCR**,: quantitative polymerase chain reaction

## References

[bibr01] Barash IA, Mathew L, Lahey M, Greaser ML, Lieber RL. (2005). Muscle LIM protein plays both structural and functional roles in skeletal muscle.. *American Journal of Physiology- Cell Physiology*.

[bibr02] Barat-Houari M, Hilliou F, Jousset F, Sofer L, Deleury E, Rocher J, Ravallec M, Galibert L, Delobel P, Feyereisen R. (2006). Gene expression profiling of *Spodoptera frugiperda* hemocytes and fat body using cDNA microarray reveals polydnavirus-associated variations in lepidopteran host genes transcript levels.. *BioMed Central Genomics*.

[bibr03] Belvin MP, Anderson KV. (1996). A conserved signaling pathway: the *Drosophila* toll-dorsal pathway.. *Annual Review of Cell and Developmental Biology*.

[bibr04] Diatchenko L, Lau Y, Campbell AP, Chenchik A, Moqadam F, Huang B, Lukyanov S, Lukyanov K, Gurskaya N, Sverdlov ED. (1996). Suppression subtractive hybridization: a method for generating differentially regulated or tissue-specific cDNA probes and libraries.. *Proceedings of the National Academy of Sciences of the USA*.

[bibr05] Dong K, Zhang D, Dahlman DL. (1996). Down-regulation of juvenile hormone esterase and arylphorin production in *Heliothis virescens* larvae parasitized by *Microplitis croceipes*.. *Archives of Insect Biochemistry and Physiology*.

[bibr06] Fort S, Wanner KW, Maleszka R. (2007). Chemosensory proteins in the honey bee: Insights from the annotated genome, comparative analyses and expressional profiling.. *Insect Biochemistry and Molecular Biology*.

[bibr07] Goldsmith MR, Shimada T, Abe H. (2005). The genetics and genomics of the silkworm, *Bombyx Mori*.. *Annual Review of. Entomology*.

[bibr08] Grogan GJ, Holland HL. (2000). The biocatalytic reactions of *Beauveria* spp.. *Journal of Molecular Catalysis B: Enzymatic*.

[bibr09] Hao Z, Kasumba I, Lehane MJ, Gibson WC, Kwon J, Aksoy S. (2001). Tsetse immune responses and trypanosome transmission: implications for the development of tsetsebased strategies to reduce trypanosomiasis.. *Proceedings of the National Academy of Sciences of the USA*.

[bibr10] Hoffmann JA. (2003). The immune response of *Drosophila*.. *Nature*.

[bibr11] Hoffmann JA, Reichhart JM. (2002). Drosophila innate immunity: an evolutionary perspective.. *Nature Immunology*.

[bibr12] Hou C, Qing G, Liu T, Mei X, Zhang R, Zhao P, Shen Z, Guo X. (2011). Differential gene expression in silkworm in response to *Beauveria bassiana* infection.. *Gene*.

[bibr13] Kucharski R, Maleszka R. (2003). Transcriptional profiling reveals multifunctional roles for transferrin in the honeybee, *Apis mellifera*.. *Journal of Insect Science*.

[bibr14] Lavine M, Strand M. (2002). Insect hemocytes and their role in immunity.. *Insect Biochemistry and Molecular Biology*.

[bibr15] Li M, Shen L, Xu A, Miao X, Hou C, Sun P, Zhang Y, Huang Y. (2005). Genetic diversity among silkworm (*Bombyx mori* L., Lep., Bombycidae) germplasms revealed by microsatellites.. *Genome*.

[bibr16] Liu F, Ling E, Wu S. (2009). Gene expression profiling during early response to injury and microbial challenges in the silkworm, *Bombyx mori*.. *Archives of Insect Biochemistry and Physiology*.

[bibr17] Liu Y, Niu BL, Weng HB, Shen WF, He LH, Qi XP, Meng ZQ. (2007). Cloning and structural analysis of MLP in the silkworm, *Bombyx mori*.. *Yi Chuan*.

[bibr18] Mine E, Izumi S, Katsuki M, Tomino S. (1983). Developmental and sex-dependent regulation of storage protein synthesis in the silkworm, *Bombyx mori*.. *Developmental Biology*.

[bibr19] Nichol H, Law JH, Winzerling JJ. (2002). Iron metabolism in insects.. *Annual Review of Entomology*.

[bibr20] Nomura KA, Arai T, Kubo T, Natori S. (1998). Molecular cloning of cDNA for p10, a novel protein that increases in the regenerating legs of *Periplaneta americana* (American cockroach).. *Insect Biochemistry and Molecular Biology*.

[bibr21] Oduol F, Xu J, Niaré O, Natarajan R, Vernick KD. (2000). Genes identified by an expression screen of the vector mosquito *Anopheles gambiae* display differential molecular immune response to malaria parasites and bacteria.. *Proceedings of the National Academy of Sciences of the USA*.

[bibr22] Sabatier L, Jouanguy E, Dostert C, Zachary D, Dimarcq JL, Bulet P, Imler JL. (2003). Pherokine-2 and -3: Two Drosophila molecules related to pheromone/odor-binding proteins induced by viral and bacterial infections.. *European Journal of Biochemistry*.

[bibr23] Shelby KS, Webb BA. (1997). Polydnavirus infection inhibits translation of specific growth-associated host proteins.. *Insect Biochemistry and Molecular Biology*.

[bibr24] Spyliotopoulos A, Gkouvitsas T, Fantinou A, Kourti A. (2007). Expression of a cDNA encoding a member of the hexamerin storage proteins from the moth *Sesamia nonagrioides* (Lef.) during diapause.. *Comparative Biochemistry and Physiology Part B: Biochemistry and Molecular Biology*.

[bibr25] Stathopoulos A, Van DM, Erives A, Markstein M, Levine M. (2002). Wholegenome analysis of dorsal-ventral patterning in the *Drosophila* embryo.. *Cell*.

[bibr26] Thompson GJ, Crozier YC, Crozier RH. (2003). Isolation and characterization of a termite transferrin gene up-regulated on infection.. *Insect Molecular Biology*.

[bibr27] Tojo S, Nagata M, Kobayashi M. (1980). Storage proteins in the silkworm, *Bombyx mori*.. *Insect Biochemistry*.

[bibr28] Tzou P, De GE, Lemaitre B. (2002). How Drosophila combats microbial infection: a model to study innate immunity and hostpathogen interactions.. *Current Opinion in Microbiology*.

[bibr29] Willott E, Wang XY, Wells MA. (1989). cDNA and gene sequence of *Manduca sexta* arylphorin, an aromatic amino acid-rich larval serum protein. Homology to arthropod hemocyanins.. *Journal of Biological Chemistry*.

[bibr30] Yoshiga T, Hernandez VP, Fallon AM, Law JH. (1997). Mosquito transferrin, an acutephase protein that is up-regulated upon infection.. *Proceedings of the National Academy of Sciences of the USA*.

[bibr31] Yun EY, Kang SW, Hwang JS, Goo TW, Kim SH, Jin BR, Kwon OY, Kim KY. (1999). Molecular cloning and characterization of a cDNA encoding a transferrin homolog from *Bombyx mori*.. *Biological Chemistry*.

[bibr32] Yun EY, Lee JK, Kwon O. (2009). Bombyx mori transferrin: genomic structure, expression and antimicrobial activity of recombinant protein.. *Developmental &Comparative Immunology*.

[bibr33] Zhu Y, Johnson T, Myers A, Kanost M. (2003). Identification by subtractive suppression hybridization of bacteria-induced genes expressed in *Manduca sexta* fat body.. *Insect Biochemistry and Molecular Biology*.

